# Supplementary figures and images for: High Fructose and High Fat Diet Impair Different Types of Memory through Oxidative Stress in a Sex- and Hormone-Dependent Manner
Source: Metabolites. 2022 Apr 12;12(4):341. doi: 10.3390/metabo12040341 (PMC9024673; doi:10.3390/metabo12040341)

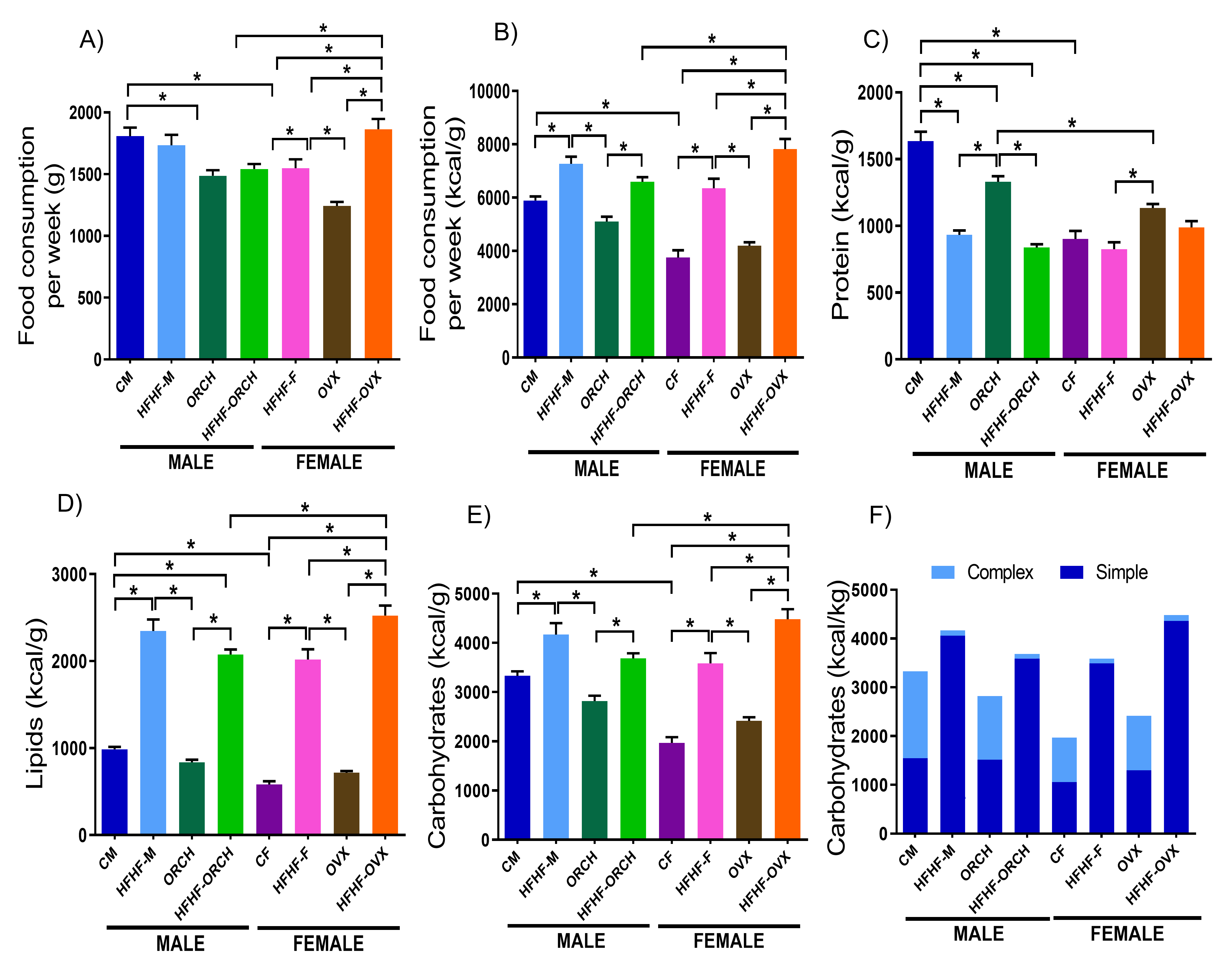

Supplement: Supplementary file 1 [file metabolites-12-00341-s001.zip › metabolites-1665368-supplementary.png]
